# PVDF/MOFs mixed matrix ultrafiltration membrane for efficient water treatment

**DOI:** 10.3389/fchem.2022.985750

**Published:** 2022-08-12

**Authors:** Lilantian Cheng, Zixun Zhou, Lei Li, Pei Xiao, Yun Ma, Fei Liu, Jian Li

**Affiliations:** ^1^ Laboratory of Environmental Biotechnology, Jiangsu Engineering Laboratory for Biomass Energy and Carbon Reduction Technology, Jiangsu Key Laboratory of Anaerobic Biotechnology, School of Environmental and Civil Engineering, Jiangnan University, Wuxi, China; ^2^ State Key Laboratory of Food Science and Technology, Science Center for Future Foods, School of Food Science and Technology, International Joint Laboratory on Food Safety, Jiangnan University, Wuxi, China

**Keywords:** ultrafiltration, MIL-53(Al), PVDF, mixed matrix membrane, antifouling

## Abstract

Polyvinylidene fluoride (PVDF), with excellent mechanical strength, thermal stability and chemical corrosion resistance, has become an excellent material for separation membranes fabrication. However, the high hydrophobicity of PVDF membrane surface normally leads a decreased water permeability and serious membrane pollution, which ultimately result in low operational efficiency, short lifespan of membrane, high operation cost and other problems. Metal-organic frameworks (MOFs), have been widely applied for membrane modification due to its large specific surface area, large porosity and adjustable pore size. Currently, numerous MOFs have been synthesized and used to adjust the membrane separation properties. In this study, MIL-53(Al) were blended with PVDF casting solution to prepare ultrafiltration (UF) membrane through a phase separation technique. The optimal separation performance was achieved by varying the concentration of MIL-53(Al). The surface properties and microstructures of the as-prepared membranes with different MIL-53(Al) loading revealed that the incorporation of MIL-53(Al) enhanced the membrane hydrophilicity and increased the porosity and average pore size of the membrane. The optimal membrane decorated with 5 wt% MIL-53(Al) possessed a pure water permeability up to 43.60 L m^−2^ h^−1^ bar^−1^, while maintaining higher rejections towards BSA (82.09%). Meanwhile, the prepared MIL-53(Al)/LiCl@PVDF membranes exhibited an excellent antifouling performance.

## 1 Introduction

Membrane separation technology, due to its high efficiency, low energy consumption, and environmental friendliness, has attracted substantial attention for advanced water treatment (Vatanpour et al., 2018; Karimi et al., 2020; Ma et al., 2020). Among different separation processes, ultrafiltration (UF), with the advantages of low operation costs and acceptable separation efficiency towards viruses, high-molecular-organics and colloidal contaminants, has been widely applied in industries such as the food, medicine, biological, environmental protection (He et al., 2022). Currently, most ultrafiltration membranes are fabricated with polymeric materials such as polyvinylidene fluoride (PVDF), polyethersulfone (PES), polypropylene (PP), and polysulfone (PS) (Kang and Cao, 2014; Vatanpour et al., 2018). Compared with other polymeric materials, PVDF have been gained noticeable attention for ultrafiltration membranes fabrication owing to its excellent mechanical strength, thermal stability and chemical resistance properties (Oskoui et al., 2019; Castro-Muñoz et al., 2021; Cheng et al., 2021). However, PVDF membranes are prone to be contaminated by organic pollutants because of its low surface energy and strong hydrophobicity properties (Cheng et al., 2021; Zuo et al., 2021). The fouling behavior of membrane will increase the mass transfer resistance during the filtration, which finally recall back the trade-off relationship between selectivity and permeability. In addition, membrane fouling normally increase the operational cost due to a frequent membrane cleaning and maintenance (Yu et al., 2020).

To improve the anti-fouling ability of the PVDF membrane, a host of methods have been proposed, such as chemical grafting (Liu C et al., 2019; Wu et al., 2019), surface modification (Liu et al., 2020; Tang et al., 2020), and blending of additives (Saini et al., 2020; Van Tran et al., 2021). Among these methods, blending of additives, especially nanomaterials, has attracted much attention due to their simple fabrication process and moderate operation conditions. Mixed matrix membranes (MMMs) prepared by incorporating nanomaterials into polymer matrices have been proven to possess the ability to reduce the membrane fouling properties and further improve the membrane permeability. For example, by blending sandwich-like GO@UiO-66 nanoparticles to the membrane casting solution, Liang et al. (2021) successfully fabricated an UF membrane with superior permeability due to the enhanced porosity and hydrophilicity. The dense hydration layer of the obtained membrane surface significantly enhanced the performance of the antifouling properties and BSA rejection. Zhu et al. (2020) embedded the rebar-like Fe_3_O_4_-palygorskite nanocomposites (MPGS) in PVDF matrix to acquire the MMMs with a better tensile strength. The flux recovery rate of the MMMs with 7.0 wt% MPGS (＞80%) was more than twice than that of the pure PVDF membrane (31.6%). Meanwhile, the obtained membrane had an outstanding antifouling property due to the high hydrophilicity of the modified membrane. Currently, an increasing number of nanomaterials have been attempted to be blended into PVDF matrix, such as metal nanoparticles (Alnairat et al., 2021), covalent organic frameworks (COFs) (Xu et al., 2020; Qian et al., 2022), carbon nanotubes (CNTs) (Ayyaru et al., 2019; Gholami et al., 2022) etc. Thus, the choose and control of nanoparticles in UF membrane is crucial important.

Metal-organic frameworks (MOFs), a well-known class of porous crystalline inorganic-organic solid materials, provide a potential for the separation application processes due to the distinguished features of structural diversity, pore-size tunability, high surface areas, and good thermal/chemical stability (Liu et al., 2013). Importantly, due to the existence of organic ligands, the MOFs exhibited excellent compatibility with polymeric matrix than traditional nanomaterials (Xie et al., 2020; Liu et al., 2021). MOF-based MMMs have been reported to show excellent separation performance including catalytic oxidation, selective permeability, and anti-fouling performance. For example, Liu et al. (2021) reported a novel ZIF-67-imbedded PVDF (ZIF-67@PVDF) mixed-matrix UF membrane fabricated by a nonsolvent-induced phase separation (NIPS) technique. The well-dispersed ZIF-67 nanoparticles exhibited excellent catalytic activity and improved the membrane porosity. To date, numerous studies have reported the application of MOF materials, including UiO-66 (Wan et al., 2020; Wang et al., 2021), ZIF-8 (Karimi et al., 2019), HKUST-1 (Yang et al., 2021), and MIL-101 (Ni et al., 2021). Among these materials, MIL-53(M) series have attracted much attention due to its chemical versatility, flexible structure, breathing feature and stability (Naeimi and Faghihian, 2017). Besides, MIL-53(Al), with the properties of permanent porosity, outstanding structural stability, and larger specific surface area, is considered as a promising adsorbent for water treatment (Liu et al., 2013). All of these merits that MIL-53 (Al) exhibited supplied the possibilities that MIL-53(Al) could be an excellent candidate for membrane fabrication.

In this work, PVDF and MIL-53(Al) are used as the polymer matrix and inorganic filler to prepare the MMMs through a phase inversion method. The effects of MOFs concentration on the morphology and physicochemical properties of the UF membrane were explored. The performance of the UF membrane was also evaluated concerning the water permeability, BSA rejections, and antifouling performance.

## 2 Experimental

### 2.1 Materials

N,N-Dimethylformamide (DMF, AR grade), aluminum nitrate nonahydrate (Al(NO_3_)_3_·9H_2_O, AR grade), and terephthalic acid (H_2_BDC, AR grade) purchased from Sigma-Aldrich (China) were adopted to synthesize MIL-53(Al). Absolute ethanol (CH_3_OH, AR grade) and lithium Chloride (LiCl, 99.0% purity) were provided by Sinopharm Chemical Reagent Co., Ltd. (Shanghai, China). Poly (vinylidene fluoride) (PVDF) was obtained from Solvey (Shanghai) Co., Ltd. Bovine serum albumin (BSA, Molecular weight=66 kDa) was purchased from Beyotime Biotechnology Co., Ltd. (Shanghai, China).

### 2.2 Synthesis of MIL-53 (Al)

**FIGURE 1:**
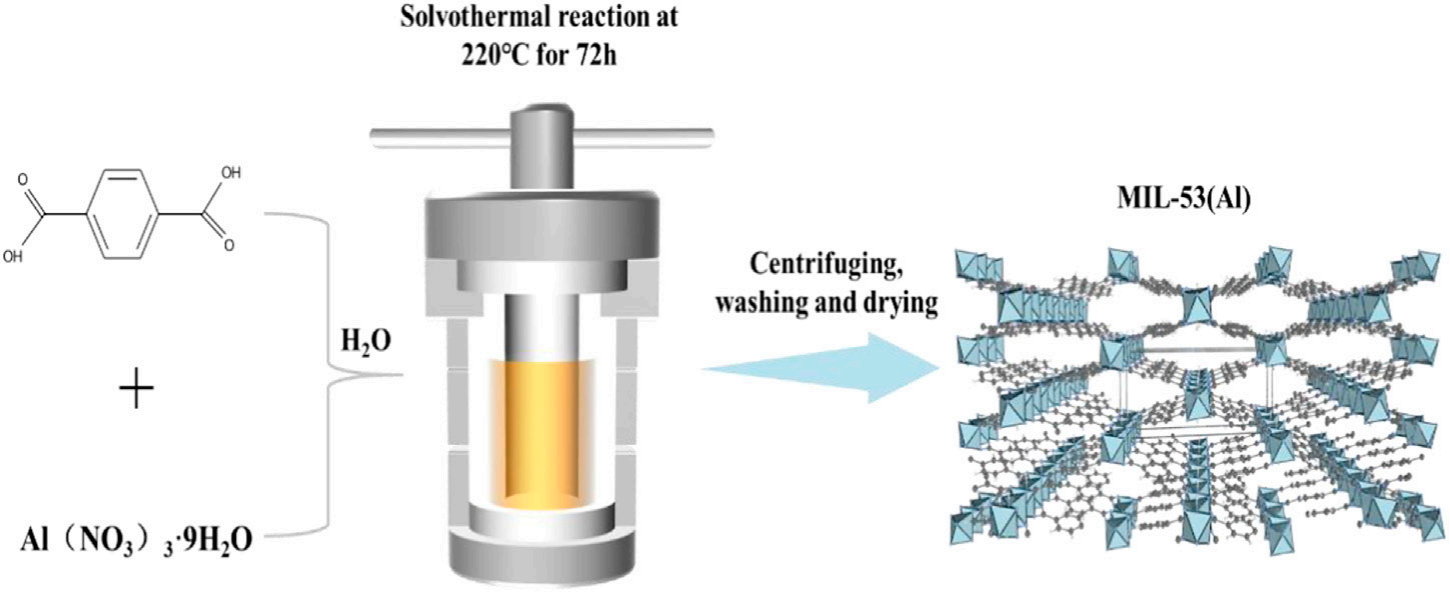
Schematic illustration of the synthesis of MIL-53 (Al) by solvothermal methods.

The method for MIL-53(Al) synthesize was conducted according to the previous study (Figure 1) (Mounfield and Walton, 2015). Firstly, Al(NO_3_)_3_·9H_2_O, H_2_BDC, and deionized (DI) water were mixed together with a molar ratio of 1:1:222. After magnetic stirring for 40 min, the as-prepared mixture was heated at 220°C for 72 h in a Teflon-lined steel autoclave. After the solvothermal reaction, the steel autoclave was gradually cooled down to room temperature (RT). The product was centrifuged and washed with DI water, DMF and ethanol, sequentially. Finally, the obtained powder products were placed into an oven and dried for 24 h at 70°C.

### 2.3 Fabrication of MIL-53(Al)/LiCl@PVDF membranes

**FIGURE 2:**
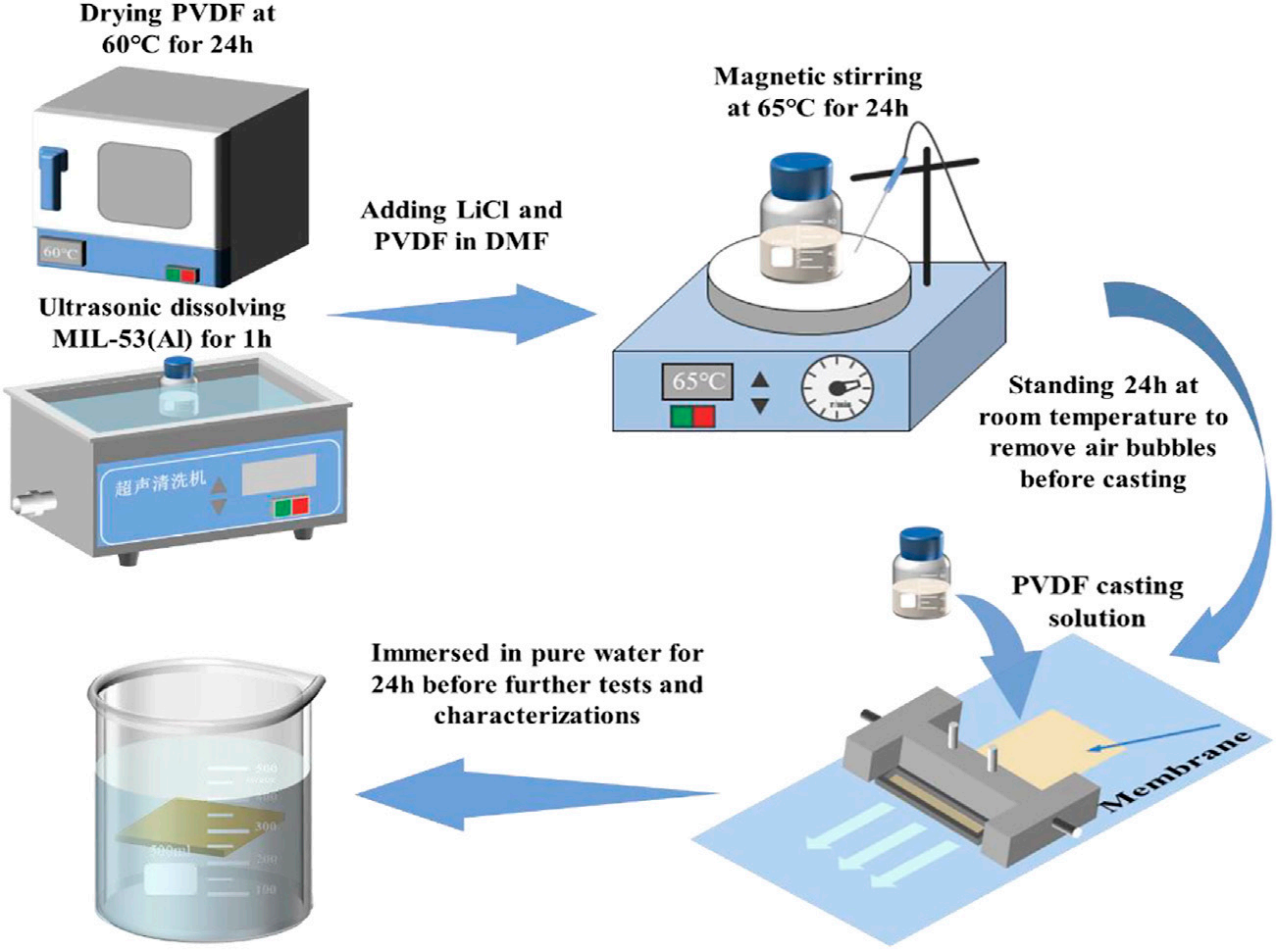
Schematic illustration of the preparation process of MIL/LiCl@PVDF membranes.

The preparation process of MIL/LiCl@PVDF membranes is shown in Figure 2. First, the as-prepared MIL-53(Al) powders were dispersed in DMF (PH = 7.0) and sonicated for 1 h to obtain a homogeneous mixed solution. After that, a certain proportion of LiCl and PVDF were added to the mixed solution and stirred for 24 h at 65°C to obtain an uniform casting solution. After defoaming for 24 h, the casting solution was poured slowly onto a clean glass plate at room temperature and the membrane was scraped by a scraping machine with speed of 100 mm/s. The obtained membrane with a thickness of 200 µM was immersed in a pure water and soaked for 24 h to remove excess organic solvent (Figure 2). The fabricated membranes were labelled as M1, M2, M3, M4, and M5, according to the quality percentage of MIL-53(Al). For comparison, the control membrane (labelled as M0) was also fabricated based on the above procedure without adding MIL-53(Al). The compositions of the casting solutions are exhibited in Table 1.

**TABLE 1:** The composition of the casting solutions.

Membrane	Compositions			
	DMF (wt%)	LiCl (wt%)	PVDF (wt%)	MIL-53 (Al) (wt%)
M	80	—	15	—
M0	80	5	15	—
M1	80	5	15	1
M2	80	5	15	3
M3	80	5	15	5
M4	80	5	15	7
M5	80	5	15	9

### 2.4 Membrane characterization

The intensity-weighted hydrodynamic diameter (D_h_) and Zeta potentials of MIL-53(Al) were measured by using a dynamic light scattering (DLS) analyzer (ZEN3600, Malvren, United Kingdom). The morphologies of the obtained membranes were observed by the scanning electron microscope (SEM, XL30 FEG, Netherlands or SU8100, Japan). X-ray diffraction (XRD) patterns of the fabricated membranes and MIL-53(Al) powders were collected by X-ray diffractometer (Rigku Ultima IV, Japan). Fourier transform infrared (FTIR, Thermo FTIR-iS10) was adopted to investigate the chemical structure of the membrane in the range of 500–4,000 cm^−1^. The contact angles of the membranes were measured by a contact angle meter (OCA20, Dataphysics Instruments, Germany) at room temperature with 2.0 µl deionized (DI) water.

The porosity and mean pore size of the membranes were measured according to a dry-wet weight method. The membrane samples were cut into rectangles with an area of 4 cm^2^ and soaked in DI water. After mopping the superficial water with filter paper, the weight of the wet membrane was recorded as W_1_. Afterwards, the wet membranes were dried at 60°C for 24 h and weighed as W_2_. Each types of membrane were tested for three times and the average value was taken. The membrane porosity (ε) was calculated by the following Eq. 1:

$$\varepsilon =\frac{{\left(W_{1}-W\right)}_{2}}{\rho_{w}A\delta }\times 100\%$$ (1)

Where W_1_ and W_2_ (kg) are the weights of the wet and dry membranes. ρ_w_ (g/cm^3^) is the density of the pure water, A (cm^2^) is the membrane area, and δ (µM) is the average membrane thickness. The mean pore radius of the membrane was calculated by the following Eq. 2 (Yong et al., 2019):

$$r_{m}=\sqrt{\frac{\left(2.9-1.75\varepsilon \right)8\eta \delta Q}{\varepsilon A\Delta P}}$$ (2)

The antifouling property of membranes was evaluated by the Flux Recovery Ratio (FRR). First, the pure water flux (J_W1_) of the membranes was measured at 0.1 MPa for 30 min. Then, the BSA aqueous solution (1 g/L) was used as the feed and the flux (J_P_) was recorded for every 10 min interval during the period of 30 min. After filtration of the BSA solution, the membranes were washed with deionized water for 30 min and the water flux of cleaned membranes (J_W2_) was measured again. FRR was calculated by the following Eq. 3:

$$\;FRR=\frac{J_{W1}}{J_{W2}}\times 100\%$$ (3)

Furthermore, to further analyze the fouling process in details, total fouling ratio (R_t_), reversible fouling ratio (R_r_) and irreversible fouling ratio (R_ir_) were calculated using the following equations:

$$R_{t}=\left(1-\frac{J_{p}}{J_{W1}}\right)\times 100\;$$ (4)

$$R_{r}=\left(\frac{J_{W2}-J_{p}}{J_{W1}}\right)\times 100\%$$ (5)

$$R_{ir}=\left(\frac{J_{W1}-J_{W2}}{J_{W1}}\right)\times 100\%=R_{t}-R_{r}$$ (6)

## 3 Results and discussions

### 3.1 The properties of MIL-53 (Al)

**FIGURE 3:**
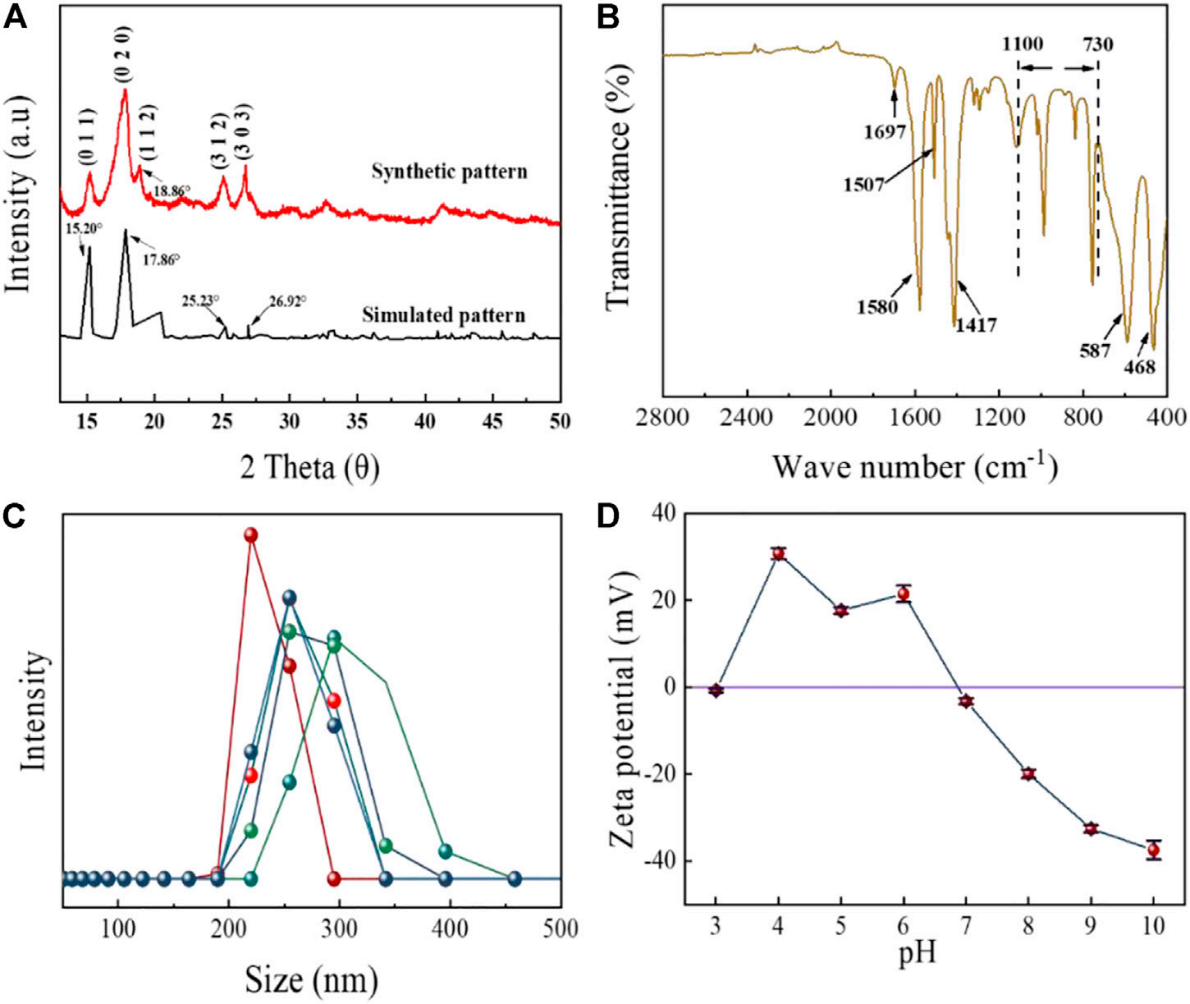
**(A)** XRD patterns, **(B)** XPS spectra, **(C)** size distribution, and **(D)** Zeta potentials of the synthesized MIL-53 (Al).

The properties of MIL-53(Al) were analyzed by XRD, FTIR, size analyzer and Zeta potential. Figure 3A shows the XRD patterns of MIL-53(Al) nanoparticles. The diffraction peaks that emerged at 2θ=15.20°, 17.86°, 25.23° and 26.92° were consistent with the literature, which indicated that the MIL-53(Al) crystals were successfully synthesized (Loloei et al., 2021; Mahdavi et al., 2021). The FTIR spectra of the synthesized MIL-53(Al) was presented in Figure 3B. The absorption peaks located around 1700–1,400 cm^−1^ are due to the presence of carboxylic functional groups (Jiang et al., 2016), the peaks at 1,507 cm^−1^and 1,580 cm^−1^ are derived by the asymmetric stretching of the carboxylate groups, while the peak located at 1,417 cm^−1^ is explained by the symmetric stretching of carboxylate groups (Jiang et al., 2016; Imanipoor et al., 2020; Aqel et al., 2021). The small peak at 1,697 cm^−1^ is derived from the stretching vibration of C=O (Venkateswarlu et al., 2020; Yang et al., 2022). In addition, the absorption peaks at 587 cm^−1^ and 468 cm^−1^ are corresponded to the Al-O bond (Liu J.-F et al., 2019). The vibration peaks between 730 and 1,100 cm^−1^ are ascribed to the stretching of C-H, which indicated the presence of organic ring in the frame structure of MOFs (Rahmani and Rahmani, 2018; Chatterjee et al., 2020). The particle size distribution of MIL-53(Al) (Figure 3C) demonstrated that the majority MIL-53(Al) were with diameter between 200 and 350 nm with an average size of 257.94 nm. The Zeta potential of MIL-53(Al) results are shown in Figure 2D. In aqueous environment, the isoelectric point (pH_PZC_) of MIL-53(Al) is 6.87. The surface of MIL-53 (Al) is positively charged at pH < pH_pzc_, while negatively charged at pH > pH_pzc_.

### 3.2 Characterization of the membranes

#### 3.2.1 The morphologies and structures

**FIGURE 4:**
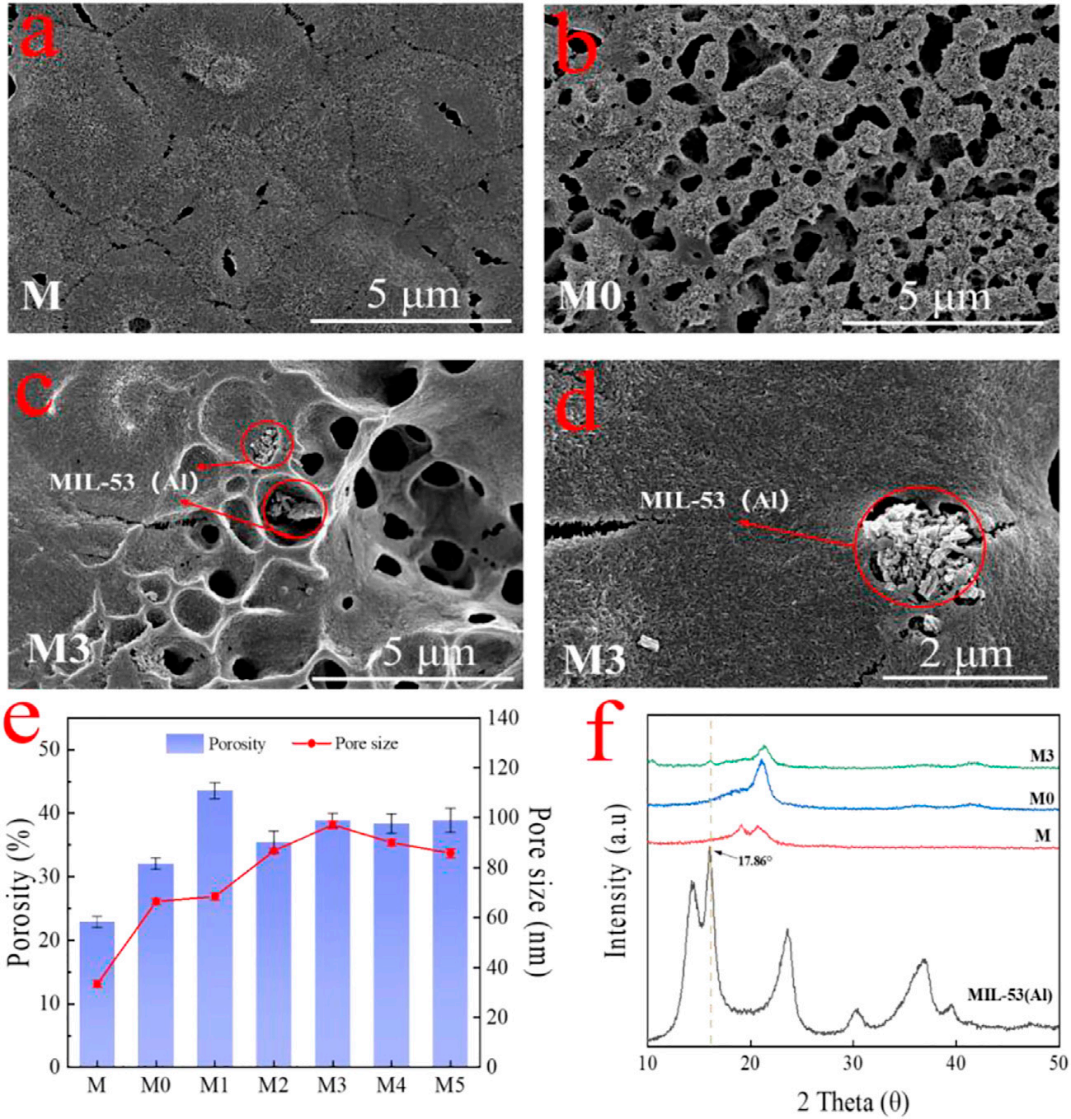
The surface morphologies of the **(A)** M membrane; **(B)** M0 membrane; **(C,D)** M3 membrane with different magnifications; **(E)** surface porosity and mean flow pore size of membranes; and **(F)** XRD pattern for the M, M0 and M3 membranes.

The surface and cross-section morphologies of the membranes were characterized by SEM. As shown in Figure 4A, the pure PVDF membrane (M) exhibited a relatively dense surface with small pores atop on it. After the addition of LiCl, a porous surface of M0 membrane (Figure 4B) can be easy identified. The pores on the membrane surface are caused by the increasing exchange rate of solvent and non-solvent due to the presence of LiCl that strongly interacted with the polymer and solvent (Zahirifar et al., 2018; Zheng et al., 2018; Pal et al., 2020). The obtained membranes modified with the addition of MIL-53 (Al) showed a larger cavities than the LiCl@PVDF membrane. When MIL-53 (Al) was introduced into the casting solution, the exchange rate of solvent and non-solvent was further accelerated, which is beneficial to increase pores size and porosity (Figure 4E) (Ayyaru et al., 2020). As it can be seen from Figures 4C,D, part of the MIL-53(Al) nanoparticles was exposed on the top of the membrane surface during the phase transformation process. The XRD patterns of the prepared membrane are shown in Figure 4F. Compared with M and M0, the characteristic diffraction peak of MIL-53 (Al) at 16.86° emerged on the M3 membrane, which confirmed the MIL-53(Al) particles were successfully incorporated inside the UF membrane.

**FIGURE 5:**
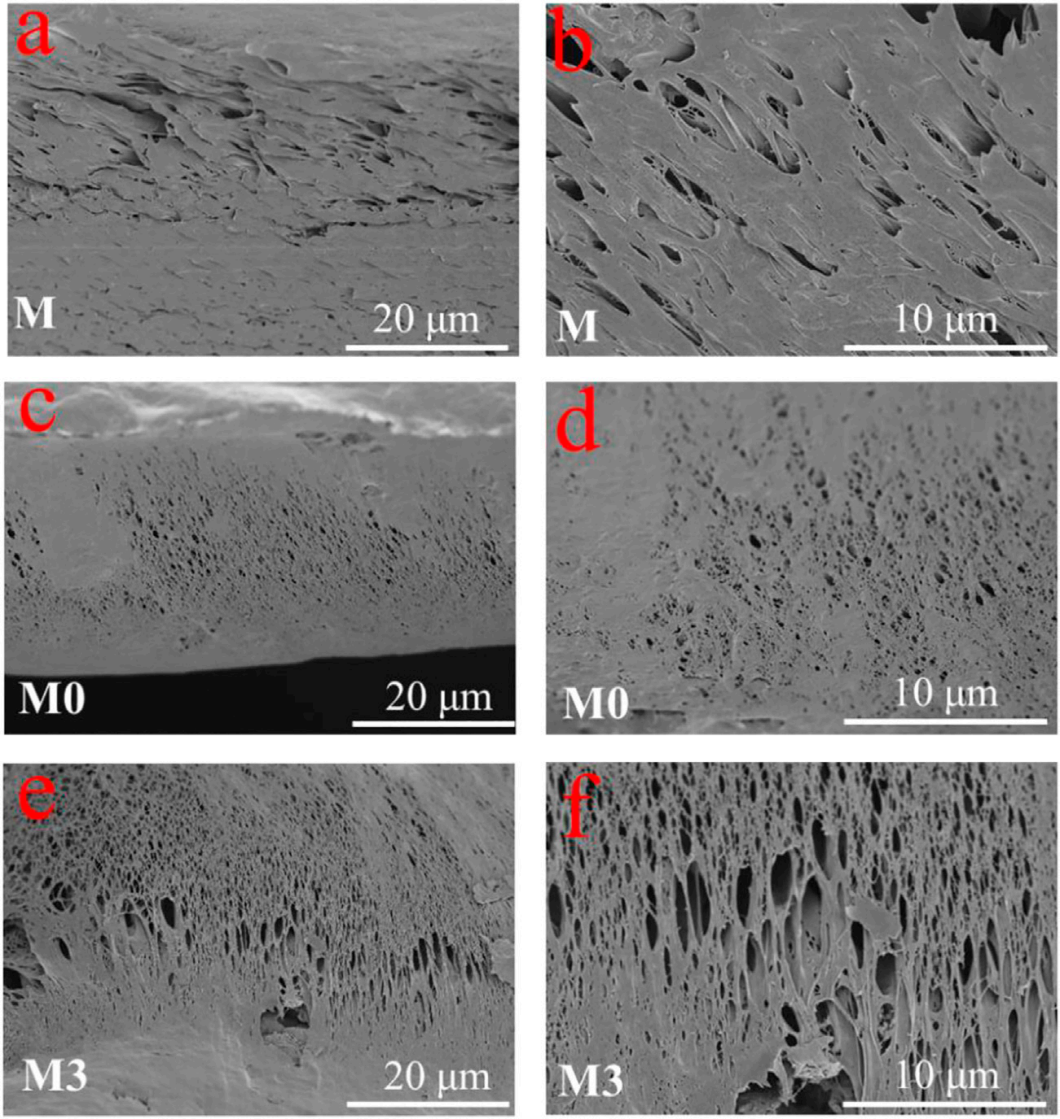
Cross-sectional SEM images of the **(A)** M membrane, **(C)** M0 membrane, **(E)** M3 membrane; and high magnification of **(B)** M membrane, **(D)** M0 membrane, **(F)** M3 membrane.

The cross-section morphologies of the membranes are shown in Figure 5. All membranes presented typical asymmetric structures with cavities and pores. The pure PVDF membrane presented a large number of small finger-like pores, which are consistent with previous researches (Karimi et al., 2020). The formation of such structure was attributed to the quick precipitation of PVDF at both the inner and the outer walls, which finally resulted in a finger-like structure (Kamaludin et al., 2022). After the addition of LiCl, the finger-like pores turned into sponge-like pores, and the thickness of the dense support layer at the bottom decreased. This variation can be attributed to the thermodynamic and kinetic effects of LiCl (Zheng et al., 2018). After further introduction of MIL-53(Al), the pores of membranes are still sponge-like, and the thickness of the dense support layer at the bottom was decreased. The transition from finger-like pores to sponge-like pores indicated that the addition of LiCl and MIL-53(Al) increases the viscosity of the casting solution and lowers the cloud point (Li et al., 2010). The sponge-like pores have a better mechanical properties than the finger-like pores (Feng et al., 2013), which is beneficial to increase the life-span of the MMMs.

**FIGURE 6:**
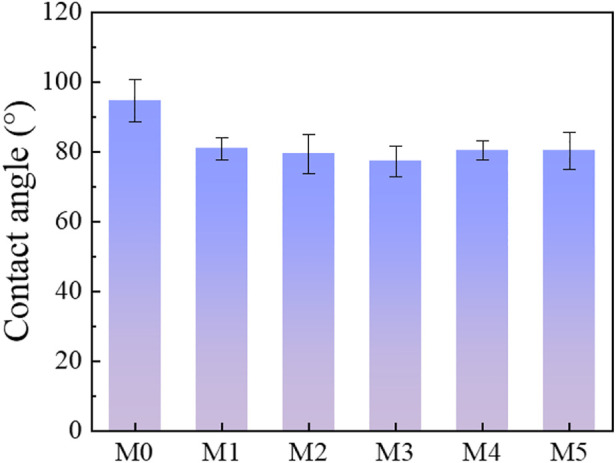
Contact angle results of membranes.

The hydrophilicity of the MIL-53(Al) decorated PVDF membranes was evaluated in Figure 6. The contact angle for the prepared MIL-53(Al)/LiCl@PVDF membranes are decreased, which indicated that the addition of MIL-53(Al) greatly enhanced the hydrophilicity of the membranes due to intrinsically hydrophilic nature of MIL-53(Al) materials. Meanwhile, the membrane surface became more hydrophilic due to the capability of the hydrophilic pores to imbibe water via capillary effects (Zhu et al., 2015). Besides, it could be observed that contact angle of M5 and M4 membranes slightly increased, as compared with TFN-3 membrane. This may be attributed to the agglomeration of MIL-53 (Al), which resulted in a rougher membrane surface and reducing the membrane hydrophilicity (Samsami et al., 2022).

#### 3.2.2 Chemical properties of the membranes

**FIGURE 7:**
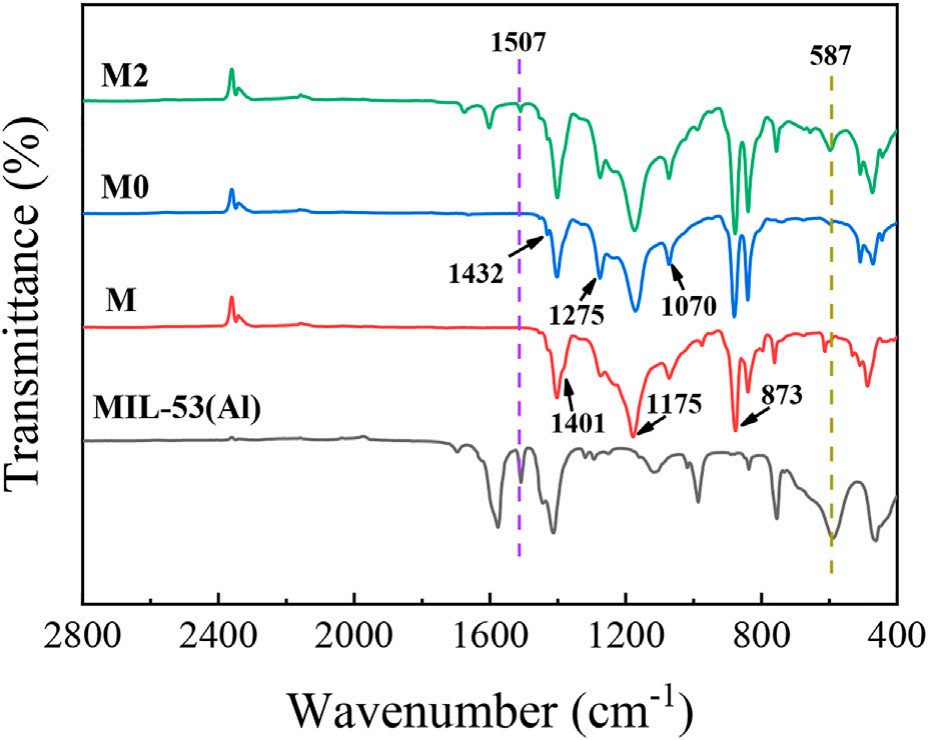
FTIR results of the M, M0 and M3 membranes.

The chemical properties of the synthetic membrane surface were analyzed by FTIR. As it can be seen from the Figure 7, three absorption peaks of PVDF at 1,401 cm^−1^, 1,175 cm^−1^ and 873 cm^−1^ are corresponded to the deformation vibrations of CH_2_, asymmetric stretching of F-C-F and skeletal vibration of C-C, respectively (Shah et al., 2021; Kachhadiya and Murthy, 2022). With the addition of inorganic salt additives of LiCl, the emerging peaks on the M0 membrane at 1,432 cm^−1^, 1275cm^−1^ and 1,070 cm^−1^ were ascribed to the β-phase polymorph (Shah et al., 2021). For M3 membrane, the new peaks at 1,507 cm^−1^ and 587 cm^−1^ are corresponding to the characteristic peaks of MIL-53(Al).

### 3.3 Separation performance of the membranes

**FIGURE 8:**
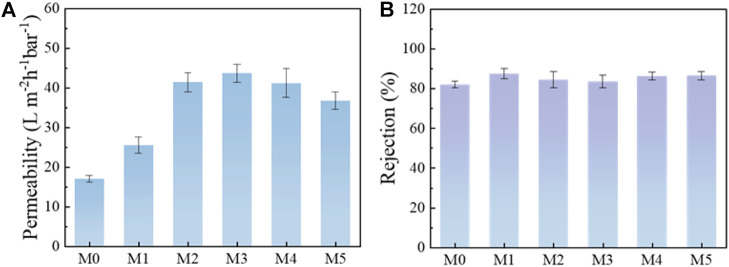
**(A)** Pure water permeability results; **(B)** rejection performance of BSA.

Based on the above analysis, the properties and structures of the membranes changed significantly after the addition of MIL-53(Al) nanoparticles, which would further affect the separation and antifouling performance of the membranes. As can be seen from Figure 8A, M0 membrane possessed a lower pure water flux of 16.98 L m^−2^ h^−1^ bar^−1^. After the incorporation of MIL-53(Al), the pure water flux of M3 membrane significantly improved to 43.60 L m^−2^ h^−1^ bar^−1^. This can be mainly explained by the addition of MIL-53(Al), which significantly improved the hydrophilicity of the membrane and reduced the mass transfer resistance. Meantime, the porous MIL-53(Al) also provided more extra transport nano-channels for water molecules. However, with excess MIL-53(Al) addition, the agglomeration of MIL-53(Al) nanoparticles could finally block the membrane pores and leading to a decrease of the pure water flux. BSA solutions was used as feed to monitor the separation experiments of the MMMs. As shown in Figure 8B, the MIL-53(Al)/LiCl@PVDF membranes were found to maintain a high rejection (>80%). Especially, for membrane with 5 wt% MIL-53(Al), M3 MMMs reached a 83.53% rejection towards BSA, while maintain an excellent pure water permeability of 43.60 L m^−2^ h^−1^ bar^−1^, simultaneously. Thus, the parameters to prepare M3 membrane was considered as the optimal modification process.

**FIGURE 9:**
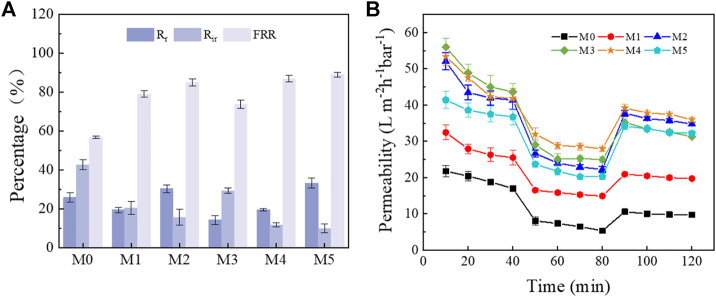
Anti-fouling performance: **(A)** water flux recovery and fouling resistance ratio of membranes; **(B)** Time-dependent flux of membranes for BSA solution filtration.

The antifouling performance of the membrane was evaluated by the filtration of BSA solution and the results were provided in Figure 9. After the filtration of BSA, the flux of M0 membrane decreased significantly, while R_r_ and R_ir_ values reached to 68.52% and 42.63%, respectively. This result indicated that BSA was adsorbed on the membrane, which caused serious membrane fouling during the filtration conditions. Compared with M0 membrane, the FRR and R_ir_ value of MIL/LiCl@PVDF ultrafiltration membrane was improved and decreased, respectively. This can be raised from the greatly improved hydrophilicity of the membrane surface after being modified with MIL-53(Al). The introduction of MIL-53(Al) could contribute to form a hydration layer and the porous MIL-53(Al) can effectively reduce the deposition of pollutants on the membrane surface due to the reduced steric hindrance effect (Xue et al., 2011). Furthermore, the R_ir_ value of the MIL/LiCl@PVDF membrane is relieved, which indicated that the antifouling ability of the ultrafiltration membrane has been improved during the filtration process.

## 4 Conclusion

In this work, a series of the LiCl@PVDF-based nanocomposite membranes with various MIL-53(Al) concentration were prepared through a phase separation technique. The results shown that the prepared MIL-53(Al)/LiCl@PVDF membranes possess an asymmetric structure with a thin dense skin layer and sponge-like sub-layer. Incorporation of hydrophilic MIL-53(Al) enhanced the membrane hydrophilicity and increased the porosity and average pore size of the membrane. At its optimism preparation conditions with 5 wt% MIL-53(Al), the obtained membrane exhibited a pure water permeability up to 43.60 L m^−2^ h^−1^ bar^−1^ and an excellent BSA rejection of 80.29%. Furthermore, the FRR value of the membranes reached up to 88.99%, which was 56.87% higher than that of the ultrafiltration membrane without the addition of MIL-53(Al), indicating that the prepared MIL-53(Al)/LiCl@PVDF membranes exhibited an excellent antifouling performance.

## Data Availability

The original contributions presented in the study are included in the article/supplementary material, further inquiries can be directed to the corresponding author.

## References

[B1] Alnairat N. Abu Dalo M. Abu-Zurayk R. Abu Mallouh S. Odeh F. Al Bawab A. (2021). Green synthesis of silver nanoparticles as an effective antibiofouling material for polyvinylidene fluoride (PVDF) ultrafiltration membrane. Polymers 13, 3683. 10.3390/polym13213683 34771241 PMC8588217

[B2] Aqel A. Alkatheri N. Ghfar A. Alsubhi A. M. ALOthman Z. A. Badjah-Hadj-Ahmed A.-Y. (2021). Preparation of value-added metal-organic frameworks for high-performance liquid chromatography. Towards green chromatographic columns. J. Chromatogr. A 1638, 461857. 10.1016/j.chroma.2020.461857 33486220

[B3] Ayyaru S. Dinh T. T. L. Ahn Y.-H. (2020). Enhanced antifouling performance of PVDF ultrafiltration membrane by blending zinc oxide with support of graphene oxide nanoparticle. Chemosphere 241, 125068. 10.1016/j.chemosphere.2019.125068 31629244

[B4] Ayyaru S. Pandiyan R. Ahn Y.-H. (2019). Fabrication and characterization of anti-fouling and non-toxic polyvinylidene fluoride-Sulphonated carbon nanotube ultrafiltration membranes for membrane bioreactors applications. Chem. Eng. Res. Des. 142, 176–188. 10.1016/j.cherd.2018.12.008

[B5] Castro-Muñoz R. González-Melgoza L. L. García-Depraect O. (2021). Ongoing progress on novel nanocomposite membranes for the separation of heavy metals from contaminated water. Chemosphere 270, 129421. 10.1016/j.chemosphere.2020.129421 33401070

[B6] Chatterjee A. Jana A. K. Basu J. K. (2020). A novel synthesis of MIL-53 (Al)@ SiO 2: an integrated photocatalyst adsorbent to remove bisphenol a from wastewater. New J. Chem. 44, 18892–18905. 10.1039/d0nj03714a

[B7] Cheng K. Zhang N. Yang N. Hou S. Ma J. Zhang L. (2021). Rapid and robust modification of PVDF ultrafiltration membranes with enhanced permselectivity, antifouling and antibacterial performance. Sep. Purif. Technol. 262, 118316. 10.1016/j.seppur.2021.118316

[B8] Feng C. Khulbe K. Matsuura T. Ismail A. (2013). Recent progresses in polymeric hollow fiber membrane preparation, characterization and applications. Sep. Purif. Technol. 111, 43–71. 10.1016/j.seppur.2013.03.017

[B9] Gholami S. Llacuna J. L. Vatanpour V. Dehqan A. Paziresh S. Cortina J. L. (2022). Impact of a new functionalization of multiwalled carbon nanotubes on antifouling and permeability of PVDF nanocomposite membranes for dye wastewater treatment. Chemosphere 294, 133699. 10.1016/j.chemosphere.2022.133699 35090853

[B10] He Y. Huang X. Li T. Lv X. Tang N. Feng C. (2022). Ultrafiltration membrane fouling control by two-stage coagulant dosing with moderate pH adjustment. Desalination 537, 115893. 10.1016/j.desal.2022.115893

[B11] Imanipoor J. Mohammadi M. Dinari M. Ehsani M. R. (2020). Adsorption and desorption of amoxicillin antibiotic from water matrices using an effective and recyclable MIL-53 (Al) metal–organic framework adsorbent. J. Chem. Eng. Data 66, 389–403. 10.1021/acs.jced.0c00736

[B12] Jiang S. Yan J. Habimana F. Ji S. (2016). Preparation of magnetically recyclable MIL-53 (Al)@ SiO2@ Fe3O4 catalysts and their catalytic performance for Friedel–Crafts acylation reaction. Catal. Today 264, 83–90. 10.1016/j.cattod.2015.10.003

[B13] Kachhadiya D. D. Murthy Z. (2022). Graphene oxide modified CuBTC incorporated PVDF membranes for saltwater desalination via pervaporation. Sep. Purif. Technol. 290, 120888. 10.1016/j.seppur.2022.120888

[B14] Kamaludin R. Abdul Majid L. Othman M. H. D. Mansur S. Sheikh Abdul Kadir S. H. Wong K. Y. (2022). Polyvinylidene Difluoride (PVDF) hollow fiber membrane incorporated with antibacterial and anti-fouling by Zinc Oxide for water and wastewater treatment. Membranes 12, 110. 10.3390/membranes12020110 35207032 PMC8878803

[B15] Kang G.-d. Cao Y.-m. (2014). Application and modification of poly (vinylidene fluoride)(PVDF) membranes–a review. J. Membr. Sci. 463, 145–165. 10.1016/j.memsci.2014.03.055

[B16] Karimi A. Khataee A. Vatanpour V. Safarpour M. (2019). High-flux PVDF mixed matrix membranes embedded with size-controlled ZIF-8 nanoparticles. Sep. Purif. Technol. 229, 115838. 10.1016/j.seppur.2019.115838

[B17] Karimi A. Khataee A. Vatanpour V. Safarpour M. (2020). The effect of different solvents on the morphology and performance of the ZIF-8 modified PVDF ultrafiltration membranes. Sep. Purif. Technol. 253, 117548. 10.1016/j.seppur.2020.117548

[B18] Li Q. Xu Z. L. Yu L. Y. (2010). Effects of mixed solvents and PVDF types on performances of PVDF microporous membranes. J. Appl. Polym. Sci. 115, 2277–2287. 10.1002/app.31324

[B19] Liang Z. Wang J. Zhang Q. Zhuang T. Zhao C. Fu Y. (2021). Composite PVDF ultrafiltration membrane tailored by sandwich-like GO@ UiO-66 nanoparticles for breaking the trade-off between permeability and selectivity. Sep. Purif. Technol. 276, 119308. 10.1016/j.seppur.2021.119308

[B20] Liu C. Wang W. Li Y. Cui F. Xie C. Zhu L. (2019). PMWCNT/PVDF ultrafiltration membranes with enhanced antifouling properties intensified by electric field for efficient blood purification. J. Membr. Sci. 576, 48–58. 10.1016/j.memsci.2019.01.015

[B21] Liu D. Yin J. Tang H. Wang H. Liu S. Huang T. (2021). Fabrication of ZIF-67@ PVDF ultrafiltration membrane with improved antifouling and separation performance for dye wastewater treatment via sulfate radical enhancement. Sep. Purif. Technol. 279, 119755. 10.1016/j.seppur.2021.119755

[B22] Liu J.-F. Mu J.-C. Qin R.-X. Ji S.-F. (2019). Pd nanoparticles immobilized on MIL-53 (Al) as highly effective bifunctional catalysts for oxidation of liquid methanol to methyl formate. Pet. Sci. 16, 901–911. 10.1007/s12182-019-0334-6

[B23] Liu J. Zhang F. Zou X. Yu G. Zhao N. Fan S. (2013). Environmentally friendly synthesis of highly hydrophobic and stable MIL-53 MOF nanomaterials. Chem. Commun. 49, 7430. 10.1039/c3cc42287a 23863860

[B24] Liu Y. Wei Y. Su J. Zhang L. Cui X. Jin L. (2020). Surface-modified PVA/PVDF hollow fiber composite membrane for air dehumidification. J. Mat. Sci. 55, 5415–5430. 10.1007/s10853-020-04373-4

[B25] Loloei M. Kaliaguine S. Rodrigue D. (2021). Mixed matrix membranes based on NH2-MIL-53 (Al) and 6FDA-ODA polyimide for CO2 separation: Effect of the processing route on improving MOF-polymer interfacial interaction. Sep. Purif. Technol. 270, 118786. 10.1016/j.seppur.2021.118786

[B26] Ma C. Hu J. Sun W. Ma Z. Yang W. Wang L. (2020). Graphene oxide-polyethylene glycol incorporated PVDF nanocomposite ultrafiltration membrane with enhanced hydrophilicity, permeability, and antifouling performance. Chemosphere 253, 126649. 10.1016/j.chemosphere.2020.126649 32268250

[B27] Mahdavi H. Karami M. Heidari A. A. kahriz P. K. (2021). Preparation of mixed matrix membranes made up of polysulfone and MIL-53 (Al) nanoparticles as promising membranes for separation of aqueous dye solutions. Sep. Purif. Technol. 274, 119033. 10.1016/j.seppur.2021.119033

[B28] Mounfield W. P. III Walton K. S. (2015). Effect of synthesis solvent on the breathing behavior of MIL-53 (Al). J. colloid interface Sci. 447, 33–39. 10.1016/j.jcis.2015.01.027 25697686

[B29] Naeimi S. Faghihian H. (2017). Application of novel metal organic framework, MIL-53 (Fe) and its magnetic hybrid: for removal of pharmaceutical pollutant, doxycycline from aqueous solutions. Environ. Toxicol. Pharmacol. 53, 121–132. 10.1016/j.etap.2017.05.007 28549314

[B30] Ni L. Zhu Y. Ma J. Wang Y. (2021). Novel strategy for membrane biofouling control in MBR with CdS/MIL-101 modified PVDF membrane by *in situ* visible light irradiation. Water Res. 188, 116554. 10.1016/j.watres.2020.116554 33128978

[B31] Oskoui S. A. Vatanpour V. Khataee A. (2019). Effect of different additives on the physicochemical properties and performance of NLDH/PVDF nanocomposite membrane. Sep. Purif. Technol. 209, 921–935. 10.1016/j.seppur.2018.09.039

[B32] Pal P. Chaurasia S. Upadhyaya S. Kumar R. Sridhar S. (2020). Development of hydrogen selective microporous PVDF membrane. Int. J. Hydrogen Energy 45, 16965–16975. 10.1016/j.ijhydene.2019.08.112

[B33] Qian M. Yan X. Chen Y. Guo X.-J. Lang W.-Z. (2022). Covalent organic framework membrane on electrospun polyvinylidene fluoride substrate with a hydrophilic intermediate layer. J. Colloid Interface Sci. 622, 11–20. 10.1016/j.jcis.2022.04.049 35490614

[B34] Rahmani E. Rahmani M. (2018). Al-based MIL-53 metal organic framework (MOF) as the new catalyst for Friedel–Crafts alkylation of benzene. Ind. Eng. Chem. Res. 57, 169–178. 10.1021/acs.iecr.7b04206

[B35] Saini B. Vaghani D. Khuntia S. Sinha M. K. Patel A. Pindoria R. (2020). A novel stimuli-responsive and fouling resistant PVDF ultrafiltration membrane prepared by using amphiphilic copolymer of poly (vinylidene fluoride) and Poly (2-N-morpholino) ethyl methacrylate. J. Membr. Sci. 603, 118047. 10.1016/j.memsci.2020.118047

[B36] Samsami S. Sarrafzadeh M.-H. Ahmadi A. (2022). Surface modification of thin-film nanocomposite forward osmosis membrane with super-hydrophilic MIL-53 (Al) for doxycycline removal as an emerging contaminant and membrane antifouling property enhancement. Chem. Eng. J. 431, 133469. 10.1016/j.cej.2021.133469

[B37] Shah V. Wang B. Li K. (2021). Blending modification to porous polyvinylidene fluoride (PVDF) membranes prepared via combined crystallisation and diffusion (CCD) technique. J. Membr. Sci. 618, 118708. 10.1016/j.memsci.2020.118708

[B38] Tang S.-H. Venault A. Hsieh C. Dizon G. V. Lo C.-T. Chang Y. (2020). A bio-inert and thermostable zwitterionic copolymer for the surface modification of PVDF membranes. J. Membr. Sci. 598, 117655. 10.1016/j.memsci.2019.117655

[B39] Van Tran T. T. Kumar S. R. Nguyen C. H. Lee J. W. Tsai H.-A. Hsieh C.-H. (2021). High-permeability graphene oxide and poly (vinyl pyrrolidone) blended poly (vinylidene fluoride) membranes: Roles of additives and their cumulative effects. J. Membr. Sci. 619, 118773. 10.1016/j.memsci.2020.118773

[B40] Vatanpour V. Ghadimi A. Karimi A. Khataee A. Yekavalangi M. E. (2018). Antifouling polyvinylidene fluoride ultrafiltration membrane fabricated from embedding polypyrrole coated multiwalled carbon nanotubes. Mater. Sci. Eng. C 89, 41–51. 10.1016/j.msec.2018.03.026 29752113

[B41] Venkateswarlu S. Reddy A. S. Panda A. Sarkar D. Son Y. Yoon M. (2020). Reversible fluorescence switching of metal–organic framework nanoparticles for use as security ink and detection of Pb2+ ions in aqueous media. ACS Appl. Nano Mat. 3, 3684–3692. 10.1021/acsanm.0c00392

[B42] Wan P. Yuan M. Yu X. Zhang Z. Deng B. (2020). Arsenate removal by reactive mixed matrix PVDF hollow fiber membranes with UIO-66 metal organic frameworks. Chem. Eng. J. 382, 122921. 10.1016/j.cej.2019.122921

[B43] Wang W. Zhang P. Shi Y. Zhang Z. Xu X. Ding P. (2021). Fabrication of *in-situ* polymerized UiO‐66/PVDF supramolecular membranes with high anti‐fouling performance. J. Appl. Polym. Sci. 138, 50519. 10.1002/app.50519

[B44] Wu Q. Tiraferri A. Wu H. Xie W. Liu B. (2019). Improving the performance of PVDF/PVDF-g-PEGMA ultrafiltration membranes by partial solvent substitution with Green solvent dimethyl sulfoxide during fabrication. ACS omega 4, 19799–19807. 10.1021/acsomega.9b02674 31788612 PMC6882131

[B45] Xie A. Cui J. Yang J. Chen Y. Lang J. Li C. (2020). Photo-Fenton self-cleaning PVDF/NH2-MIL-88B (Fe) membranes towards highly-efficient oil/water emulsion separation. J. Membr. Sci. 595, 117499. 10.1016/j.memsci.2019.117499

[B46] Xu W. Sun X. Huang M. Pan X. Huang X. Zhuang H. (2020). Novel covalent organic framework/PVDF ultrafiltration membranes with antifouling and lead removal performance. J. Environ. Manag. 269, 110758. 10.1016/j.jenvman.2020.110758 32560988

[B47] Xue Z. Wang S. Lin L. Chen L. Liu M. Feng L. (2011). A novel superhydrophilic and underwater superoleophobic hydrogel‐coated mesh for oil/water separation. Adv. Mat. 23, 4270–4273. 10.1002/adma.201102616 22039595

[B48] Yang C. P. Hu C. Y. Jiang Z. W. Xiao S. Y. Wang X. Y. Huang C. Z. (2022). Facile synthesis of porphyrin-MOFs with high photo-Fenton activity to efficiently degrade ciprofloxacin. J. Colloid Interface Sci. 622, 690–699. 10.1016/j.jcis.2022.04.104 35533483

[B49] Yang S. Tang R. Dai Y. Wang T. Zeng Z. Zhang L. (2021). Fabrication of cellulose acetate membrane with advanced ultrafiltration performances and antibacterial properties by blending with HKUST-1@ LCNFs. Sep. Purif. Technol. 279, 119524. 10.1016/j.seppur.2021.119524

[B50] Yong M. Zhang Y. Sun S. Liu W. (2019). Properties of polyvinyl chloride (PVC) ultrafiltration membrane improved by lignin: Hydrophilicity and antifouling. J. Membr. Sci. 575, 50–59. 10.1016/j.memsci.2019.01.005

[B51] Yu H. Li X. Chang H. Zhou Z. Zhang T. Yang Y. (2020). Performance of hollow fiber ultrafiltration membrane in a full-scale drinking water treatment plant in China: A systematic evaluation during 7-year operation. J. Membr. Sci. 613, 118469. 10.1016/j.memsci.2020.118469

[B52] Zahirifar J. Karimi-Sabet J. Moosavian S. M. A. Hadi A. Khadiv-Parsi P. (2018). Fabrication of a novel octadecylamine functionalized graphene oxide/PVDF dual-layer flat sheet membrane for desalination via air gap membrane distillation. Desalination 428, 227–239. 10.1016/j.desal.2017.11.028

[B53] Zheng L. Wang J. Yu D. Zhang Y. Wei Y. (2018). Preparation of PVDF-CTFE hydrophobic membrane by non-solvent induced phase inversion: Relation between polymorphism and phase inversion. J. Membr. Sci. 550, 480–491. 10.1016/j.memsci.2018.01.013

[B54] Zhu J. Zhou S. Li M. Xue A. Zhao Y. Peng W. (2020). PVDF mixed matrix ultrafiltration membrane incorporated with deformed rebar-like Fe3O4–palygorskite nanocomposites to enhance strength and antifouling properties. J. Membr. Sci. 612, 118467. 10.1016/j.memsci.2020.118467

[B55] Zhu L. Yu H. Zhang H. Shen J. Xue L. Gao C. (2015). Mixed matrix membranes containing MIL-53 (Al) for potential application in organic solvent nanofiltration. RSC Adv. 5, 73068–73076. 10.1039/c5ra10259f

[B56] Zuo C. Wang L. Tong Y. Shi L. Ding W. Li W. (2021). Co-deposition of pyrogallol/polyethyleneimine on polymer membranes for highly efficient treatment of oil-in-water emulsion. Sep. Purif. Technol. 267, 118660. 10.1016/j.seppur.2021.118660

