# Corrigendum: PVDF/MOFs mixed matrix ultrafiltration membrane for efficient water treatment

**DOI:** 10.3389/fchem.2022.1067578

**Published:** 2022-10-24

**Authors:** Lilantian Cheng, Zixun Zhou, Lei Li, Pei Xiao, Yun Ma, Fei Liu, Jian Li

**Affiliations:** ^1^ Laboratory of Environmental Biotechnology, Jiangsu Engineering Laboratory for Biomass Energy and Carbon Reduction Technology, Jiangsu Key Laboratory of Anaerobic Biotechnology, School of Environmental and Civil Engineering, Jiangnan University, Wuxi, China; ^2^ State Key Laboratory of Food Science and Technology, Science Center for Future Foods, School of Food Science and Technology, International Joint Laboratory on Food Safety, Jiangnan University, Wuxi, China

**Keywords:** ultrafiltration, MIL-53(Al), PVDF, mixed matrix membrane, antifouling

In the published article, there was an error in the author list, and author Zixun Zhou was erroneously excluded. The corrected author list appears above.

In the published article, an author name was incorrectly written as Xiao Pei. The correct name is “Pei Xiao”.

The authors apologize for these errors and state that this does not change the scientific conclusions of the article in any way. The original article has been updated.

